# Free complement and complement containing extracellular vesicles as potential biomarkers for neuroinflammatory and neurodegenerative disorders

**DOI:** 10.3389/fimmu.2022.1055050

**Published:** 2023-01-20

**Authors:** Marlies Burgelman, Pieter Dujardin, Charysse Vandendriessche, Roosmarijn E. Vandenbroucke

**Affiliations:** ^1^ VIB Center for Inflammation Research, VIB, Ghent, Belgium; ^2^ Department of Biomedical Molecular Biology, Ghent University, Ghent, Belgium

**Keywords:** complement, extracellular vesicle (EV), biomarker, neuroinflammation, Alzheimer’s disease, multiple sclerosis

## Abstract

The complement system is implicated in a broad range of neuroinflammatory disorders such as Alzheimer’s disease (AD) and multiple sclerosis (MS). Consequently, measuring complement levels in biofluids could serve as a potential biomarker for these diseases. Indeed, complement levels are shown to be altered in patients compared to controls, and some studies reported a correlation between the level of free complement in biofluids and disease progression, severity or the response to therapeutics. Overall, they are not (yet) suitable as a diagnostic tool due to heterogeneity of reported results. Moreover, measurement of free complement proteins has the disadvantage that information on their origin is lost, which might be of value in a multi-parameter approach for disease prediction and stratification. In light of this, extracellular vesicles (EVs) could provide a platform to improve the diagnostic power of complement proteins. EVs are nanosized double membrane particles that are secreted by essentially every cell type and resemble the (status of the) cell of origin. Interestingly, EVs can contain complement proteins, while the cellular origin can still be determined by the presence of EV surface markers. In this review, we summarize the current knowledge and future opportunities on the use of free and EV-associated complement proteins as biomarkers for neuroinflammatory and neurodegenerative disorders.

## 1 Introduction

The diagnosis for disorders of the central nervous system (CNS), including neurodegenerative and neuroinflammatory diseases, is still challenging (1). Because of their rising prevalence, high heterogeneity and the urge for early and accurate disease detection, the need for reliable biomarkers for diagnosis, prognosis or treatment response is high. Since dysregulation of the complement system is a common pathologic feature in several CNS diseases (2–7), a lot of research has been conducted to investigate its biomarker potential. Complement proteins are indispensable during development of the nervous system, more specifically during synaptic pruning and axonal growth (8–12). However, several studies have shown that a broad list of CNS diseases, including Alzheimer’s disease (AD) and multiple sclerosis (MS), are associated with aberrant, complement-mediated synapse elimination (5, 10, 11). Furthermore, expression and activation of complement proteins is often observed in disease-associated CNS lesions including AD and MS plaques (2, 13–16). Most importantly, in regard to biomarker research, the level of several complement proteins in biofluids is often found to be altered in CNS disease patients compared to (healthy) controls. In this review, we will discuss the potential and the limitations of complements proteins as biomarkers in neuroinflammatory and neurodegenerative diseases, focusing on AD and MS.

## 2 The complement system

**Figure 1:**
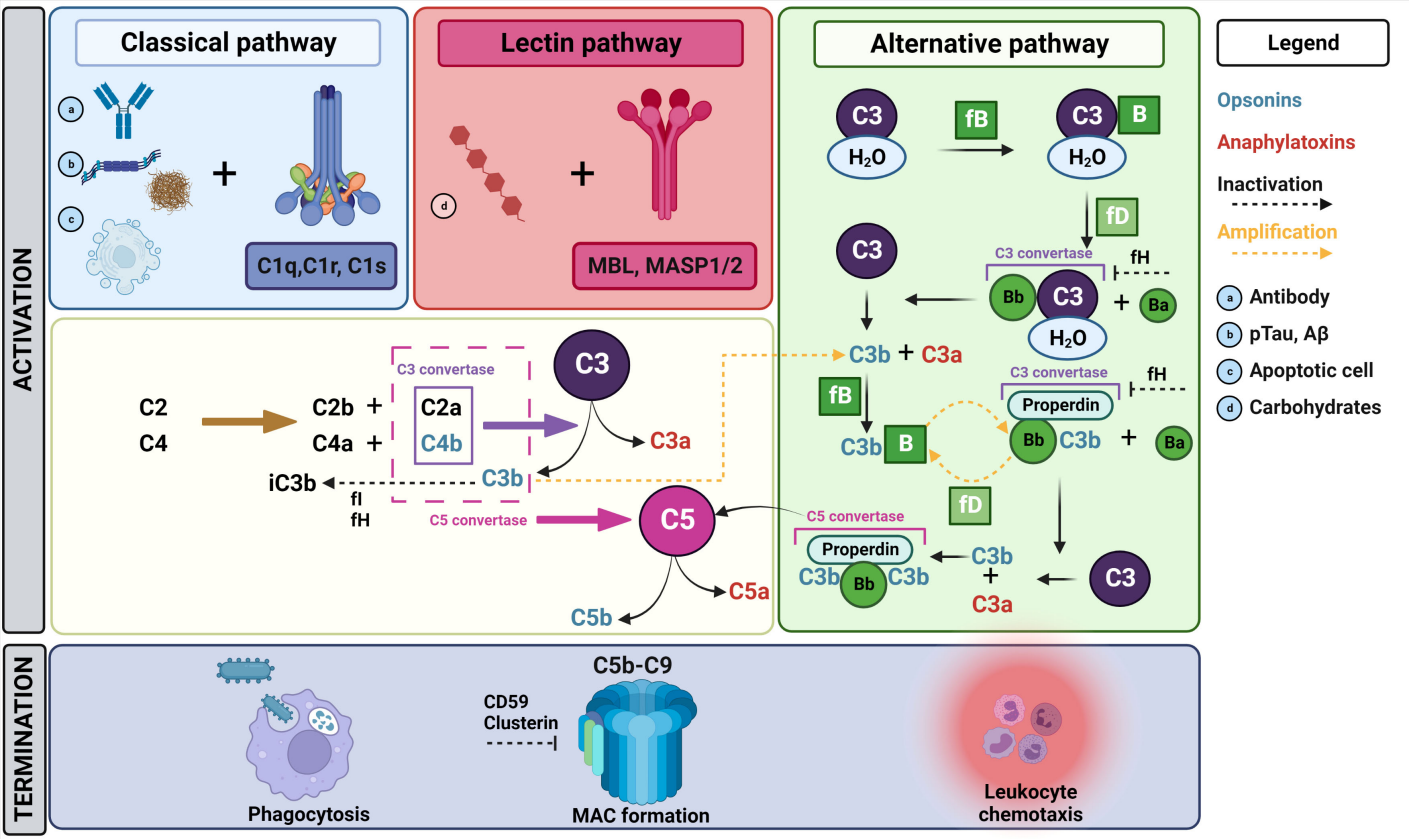
Overview of the complement cascade. The complement cascade can be activated *via* three different pathways. In the classical pathway, the C1 complex (C1r, C1s, C1q) recognizes antibody complexes **(A)** bound to pathogens, polyanionic molecules such as pTau and Aβ **(B)** or damage-associated patterns on apoptotic cells **(C)**. Following C1 activation, C2 and C4 will be converted to C2a/b and C4a/b, respectively. C2a and C4b complex together and form a C3 convertase, which will cleave C3 to create C3a and C3b. Subsequently, C3b will complex with C2aC4b to form C5 convertase, which will lead to the conversion of C5 into C5a and C5b. Finally, C5b will initiate the formation of the MAC complex by recruiting C6, C7, C8 and C9, ultimately resulting into target lysis. Several other activation products (C3b, C4b) formed during the complement pathway enhance phagocytosis *via* opsonizing the target. The lectin pathway is activated *via* the recognition of microbial carbohydrates **(D)** including MBL, resulting in the activation of MASP1/2. Thereafter, the same cascade steps as described above for C3 and C5 conversion are initiated. On the contrary, the alternative pathway is constitutively active at low level for constant scanning of cells. Here, a limited number of C3 molecules is hydrolyzed, which exposes a new binding site for fB. Subsequently, fD will cleave fB-bound C3 resulting in Bb-bound hydrolyzed C3, which functions as a C3 convertase resulting in C3a/C3b production. Thereafter, C3b will again react with fB and properdin. The resulting complex consisting of properdin, C3b and Bb also functions as a C3 convertase, which will again cleave C3 into C3b and C3a. Eventually, properdin and Bb will capture two C3b molecules to form a C5 convertase. By this, C5 can be cleaved, resulting in MAC formation as described above. As indicated in this figure, several amplification loops are present within the complement pathways. There are also different regulating factors that influence or inhibit certain parts of the cascade. For example, fH acts as inhibitor for the C3 convertases in the alternative pathway and acts as a cofactor for fI, which is in turn a regulator for the degradation of several activation products including the inactivation of C3b into iC3b. Additionally, clusterin acts as an inhibitor of MAC formation. mannan-binding lectin serine protease 1/2 (MASP1/2), mannose binding lectin (MBL), phosphorylated tau (pTau), amyloid-β (Aβ), factor I (fI), factor H (fH), factor B (fB), membrane attack complex (MAC). Figure created in Biorender and content in accordance with Dalakas et al. (3), Ricklin et al. (19) and Schartz et al. (5).

As an essential part of the innate immune system, the complement system ensures rapid recognition and clearance of pathogens or danger-associated signals (5, 17, 18). In addition, the system also coordinates adaptive immune functions (3, 18). The complement system comprises more than 40 proteins and embodies a complex set of interactions (5, 18). Overall, complement activation can be achieved *via* three pathways, which are summarized in **Figure 1** and are extensively reviewed by others (3, 5, 19, 20). The classical and lectin pathways show a similar course, however their way of activation differs (3, 5, 19). The classical pathway is triggered *via* binding of the C1 complex to the Fc domain of antibodies present in pathogen-immune complexes, or *via* its interaction with apoptotic cells or polyanionic molecules such as phosphorylated tau or amyloid-beta (Aβ) fibrils (3, 5, 19). In the lectin pathway, activation happens *via* the recognition of microbial carbohydrates by pattern binding proteins, including mannose-binding lectin (MBL), resulting in the activation of mannan-binding lectin serine protease 1 (MASP1) and MASP2 (3, 5, 19). After activation, both the classical and lectin pathway mediate C4 and C2 conversion into C4a/C4b and C2a/C2b, respectively. Subsequently, C2a and C4b complex together form the C3 convertase (C4b2a). In contrast, the alternative pathway acts differently, as hydrolyzed C3 (C3_H2O_) and factor B (fB) are cleaved by factor D (fD) and form a solvent-based C3 convertase (C3_H2O_Bb) (3, 19). The latter pathway is constantly active at low level to scan cells for alerting signals (19). Eventually, all complement activation pathways lead to the cleavage of the central complement protein C3, resulting in C3a/C3b formation (3, 5, 19). Since C3b also forms a C3 convertase together with cleaved factor B (C3bBb), a vicious cycle of C3 cleavage is formed that efficiently amplifies the response (3, 19). Subsequently, an additional C3b binds to the C3 convertase (C4b2a3b or C3bBb3b) to form C5 convertase, by which C5 is converted into C5a/C5b. These effector molecules are classified as opsonins (C3b, C5b) and anaphylatoxins (C3a, C5a) (3, 5, 19). On the one hand, opsonins will mediate efficient target elimination *via* the formation of the C5b-C9 complex, also known as the membrane attack complex (MAC) or the terminal complement complex (TCC), to mediate target lysis. On the other hand, opsonins tag the target to enhance phagocytosis. Additionally, anaphylatoxins induce leukocyte chemotaxis.

## 3 The potential of free complement proteins as biomarkers

### 3.1 Free complement as biomarker in AD

The early diagnosis of AD patients is crucial for better patient care, the timely administration of potential treatments and enrolment in clinical trials. The present reliance on clinical cognitive and behavioral testing, brain scans and medical history does not fulfill this necessity, with definitive diagnosis only possible postmortem. Unfortunately, the identification of early biomarkers is an arduous task as brain changes are estimated to already occur twenty years before symptoms arise (21, 22) together with a large individual variability in starting age, symptoms, and the speed of disease progression. It is thus of great importance to further unravel the early molecular changes to identify novel diagnostic tools. Currently, the most predictive AD biomarker is the combination of lowered levels of amyloid-beta 42 (Aβ_42_) with increased total tau (tTau) and phosphorylated tau (pTau) levels in the cerebrospinal fluid (CSF). Although promising, their levels stagnate after disease onset and do not allow staging from mild cognitive impairment (MCI) to AD dementia (23, 24). Furthermore, CSF collection by lumbar puncture is invasive and not frequently performed for AD diagnosis as is the case for MS as discussed further. As opposed to CSF, plasma AD biomarkers are less established, and the availability is still limited. Hence, additional biomarkers to improve preclinical diagnosis, staging and prognosis are necessary. The growing evidence on the involvement of the complement system in AD has brought attention on its use as such a biomarker. For example, polymorphisms in multiple complement genes are associated with increased AD risk (25–29). Furthermore, C1q is activated by Aβ and reciprocally promotes Aβ aggregation (30–33). Similarly, C1q is activated by tau and induces microglial engulfment of excitatory synapses (34, 35). A broad range of preclinical studies making use of complement inhibitors and/or knock-out (KO) mouse models also indicate that complement dysregulation is not a secondary reaction to pathology but a disease-driving force (2), for instance by mediating aberrant synaptic pruning (12, 36, 37). Consequently, complement proteins could have early biomarker potential, and numerous efforts have been made to measure and compare their levels in CSF and plasma in MCI and AD patients and/or different control groups.

#### 3.1.1 Complement proteins levels in CSF and their biomarker potential

Studies on CSF complement levels are generally consistent with either increased or unchanged levels in AD (**Table 1**). More specifically, an increase in the CSF levels of C1s (38), C1q (38), C4 (45), clusterin (39, 48, 49), complement receptor 1 (CR1) (45) and factor H (fH) (41) has been reported in AD patients compared to healthy controls (HC) and/or MCI. Conversely, unchanged levels were shown for C1q (39, 40), clusterin (50) and fH (39, 44, 46). For the central complement protein C3, the outcomes in case-control studies are most inconsistent. On one hand, we found four studies reporting elevated C3 levels in the CSF of AD patients compared to HC (41–43) or MCI (45). On the other hand, C3 concentration was unaltered in two other studies (44, 46), in which the cohort sizes were two to three times larger than in the discussed studies showing increased levels. Notably, a recent meta-analysis combining clinical data from peer-reviewed articles in close to three thousand records demonstrated increased C3 CSF levels (39) supporting the positive studies. However, the two cited studies showing unchanged C3 levels were not included in this meta-analysis, which may have skewed the comparison. Next, one study reported significantly elevated CSF C4 levels in AD patients compared to HC, and an increased trend compared to MCI (45). To our knowledge, no studies reported a decrease in complement levels in the CSF of AD/MCI patients.

**Table 1:** Overview of changes and biomarker potential of complement proteins in the cerebrospinal fluid (CSF) of mild cognitive impairment (MCI) and Alzheimer’s disease (AD) patients compared to a control group.

Cerebrospinal fluid			
Protein	Comparison	Change	Ref.
C1s	AD *vs* HC	↑	(38)
C1q	AD *vs* HC	=	(39, 40)
		↑	(38)
	MCI *vs* HC	=	(40)
	NA	+ correlation with tTau	(40)
C3	AD *vs* HC	↑	(39, 41–43)
		+ correlation with cognitive impairment (MMSE) in AD	(41, 42)
	AD *vs* MCI	=	(44)
		↑	(45)
	AD *vs* MCI *vs* HC	= - correlation with cognitive decline progression (ADAS-Cog) in MCI	(46)
		+ interaction between C3 levels and APOE E4 alleles on CSF amyloid and tau	(47)
	AD *vs* MCI	↑	(45)
C4	AD *vs* HC	↑	(45)
	AD *vs* MCI	(↑)	(45)
Clusterin	AD *vs* HC	↑	(39, 48, 49)
	AD *vs* MCI *vs* HC	= + correlation with Tau and pTau	(50)
	NA	+ correlation with Aβ-associated atrophy in non-demented elderly	(51)
CR1	AD/MCI-AD *vs* MCI/HC	↑	(45)
Factor H	AD *vs* HC	=	(39)
		↑	(41)
		+ correlation with cognitive impairment (MMSE) in AD	(41)
	AD *vs* MCI	=	(44)
	AD *vs* MCI *vs* HC	= - correlation with cognitive decline progression (ADAS-Cog) and lateral ventricular volume in MCI	(46)

The characteristics of the patient groups that were compared are indicated in the ‘comparison’ column. The ‘change’ column indicates which level alteration was observed for the respective complement component. Symbols: ↑ significant increase, (↑) increased, unsignificant trend, = unchanged. Amyloid-beta (Aβ), Alzheimer’s disease (AD), Alzheimer's Disease Assessment Scale-Cognitive Subscale (ADAS-Cog), healthy control (HC), mild cognitive impairment (MCI), mini-mental state examination (MMSE), not applicable (NA), total tau (tTau).

Although significant differences in multiple CSF complement components were found between diagnostic groups, none was reported to show adequate capability for classifying AD *versus* MCI/HC patients. More specifically, Daborg et al. showed that adding the complement proteins CR1, C3 and C4 to a multivariate constructed receiver operating characteristic (ROC) curve for the core biomarkers tTau, pTau and Aβ_42_ did not improve the area under the curve (AUC) (45). In the study of Toledo et al. the addition of C3, fH or C3:fH ratios did not improve the performance of tTau:Aβ_42_ in classifying AD subjects or MCI subjects *versus* HC (AUC 0.84 regardless of C3 and/or fH inclusion) (46). Similarly, Wang et al. showed that none of the ROC calculations using C3 or fH alone yielded acceptable sensitivity and/or specificity (41). The only exception was when the value of C3:Aβ_42_ and fH:Aβ_42_ ratios was also evaluated for AD *versus* control, where high sensitivity and specificity were achieved (C3:Aβ42, sensitivity 92.1% and specificity 76.9%; fH:Aβ_42_, sensitivity 92.1% and specificity 80.2%). Finally, Brosserson et al. indicated that CSF C1q levels in AD patients *versus* HC are not diagnostic with a discriminative power (equally weighted sensitivity and specificity) around 60% (40).

Taken together, CSF complement levels seem unsuitable as diagnostic AD biomarkers. Nevertheless, they could have potential as predictor for disease severity. A longitudinal analysis of MCI patients indicated a negative correlation between C3 and fH levels and cognitive decline progression (46) but this was not reflected by lower levels in AD/MCI patients in cross-sectional analyses. In addition, lower fH levels were also shown to correlate with increased lateral ventricular volume in MCI patients, which represents a marker of disease progression (52). As opposed to the inverse correlation between complement levels and cognitive impairment in MCI, a positive correlation is observed in AD patients. Indeed, in a cohort of AD patients with CSF hemoglobin concentration less than 200 ng/ml, a significant correlation between lower mini-mental state examination (MMSE) score and increased concentrations of CSF C3 and fH was reported (41). In addition, another study mentions that CSF C3 was positively correlated with cognitive impairment in their AD cohort, but it should be noted that the data and analysis could not be found in the report (42). Finally, it has been reported that C1q, C3 and clusterin are also associated with higher tTau levels (40, 47, 50) and that clusterin correlates with Aβ-associated atrophy in non-demented elderly (51).

#### 3.1.2 Complement proteins levels in the blood and their biomarker potential

Because of the accessibility of plasma- over CSF-based biomarkers, their identification and potential use has been explored extensively (**Table 2**). However, since the blood is not in direct contact with the diseased brain as is the case for CSF, combined with its complexity and heterogeneity, the identification of reproducible plasma biomarkers is challenging. Moreover, the majority of soluble complement proteins originate from the liver (84) which could mask brain-derived complement perturbations. Accordingly, AD plasma biomarker studies are more heterogenous and conflicting in comparison to CSF studies.

**Table 2:** Overview of changes and biomarker potential of complement proteins in the plasma of Alzheimer’s disease (AD) and mild cognitive impairment (MCI) patients compared to a control group.

Plasma			
Protein	Comparison	Change	Ref.
C1q	AD *vs* HC	(↑)	(39)
C1R	AD *vs* HC	(↑)	(53)
C1s	AD *vs* HC	=	(54)
	MCI convertors *vs* MCI non-convertors	=	(54)
C3	AD *vs* HC	=	(39)
		trend for + correlation with cognitive decline (MMSE) in combined group of AD and MCI	(55)
		↑	(55–57)
		(↑)	(53)
	aMC *vs* NC	↑	(58)
	MCI *vs* HC	↑	(57, 59)
	sMC *vs* NC	↑	(58)
	AD *vs* MCI	↓	(59)
	NA	- correlation with AD risk. Risk amplified in APOE ϵ44 carriers	(60)
iC3b	AD *vs* HC	=	(54)
	MCI convertors *vs* MCI non-convertors	=	(54)
C4	AD *vs* HC	=	(39, 53)
		↑	(56, 59)
C4a	AD *vs* HC	↑	(61)
		(↑)	(39)
C4d	AD *vs* HC	=	(54)
	MCI convertors *vs* MCI non-convertors	=	(54)
	aMC *vs* NC	↑	(58)
	sMC *vs* NC	↑	(58)
C6	aMC *vs* NC	↓	(58)
	sMC *vs* NC	↓	(58)
C9	AD *vs* HC	=	(54)
		(↑)	(39)
	MCI convertors *vs* MCI non-convertors	=	(54)
TCC	AD *vs* HC	=	(54)
	MCI convertors *vs* MCI non-convertors	↓	(54)
C1 inhibitor	AD *vs* HC	↓	(53)
	NA	- correlation with rosiglitazone drug efficacy in AD patients as defined by change in ADAS-Cog score	(62)
Clusterin	AD *vs* HC	=	(39, 48, 50, 63)
		↑	(54, 56)
	AD *vs* HC *vs* LBD *vs* depr *vs* FTD *vs* VD *vs* PD	=	(64)
	MCI convertors *vs* MCI non-convertors	↑	(54)
	MCI *vs* HC	↑	(50)
	NA	- correlation with cognitive decline rate in AD (MMSE)	(50)
		+ correlation with severity of AD (MMSE)	(63, 65)
		+ correlation with entorhinal cortex atrophy, disease severity (MMSE) and rapid clinical progression in AD	(66)
		+ correlation with cognitive decline rate in MCI (MMSE)	(50)
		+ correlation with decreased brain atrophy rate in MCI	(67)
		↑ AD risk	(65)
		↓AD risk in younger old persons (59–61, 63, 68–73) ↑ AD risk in older persons (74–83)	(72)
		↑ risk for progression from MCI to AD	(50)
CR1	AD *vs* HC	↓	(59)
Factor B	AD *vs* HC	=	(53)
		(↓)	(39)
Factor Bb	AD *vs* HC	=	(54)
	MCI convertors *vs* MCI non-convertors	=	(54)
Factor H	AD *vs* HC	=	(39, 53, 54, 70)
		↑	(56, 71)
	MCI convertors *vs* MCI non-convertors	=	(54)
	MCI *vs* HC	↑	(57, 59)
	NA	- correlation with rosiglitazone drug efficacy in AD patients (ADAS-Cog)	(62)
		+ correlation with disease severity (MMSE)	(71)
Factor I	AD *vs* HC	=	(54)
	AD *vs* MCI	↑	(59)
	MCI convertors *vs* MCI non-convertors	↓	(54)

The characteristics of the patient groups that were compared are indicated in the ‘comparison’ column. The ‘change’ column indicates which level alteration was observed for the respective complement component. Symbols: ↑ significant increase, (↑) increased, unsignificant trend, ↓ significant decrease, = unchanged. Alzheimer’s disease (AD), Alzheimer’s Disease Assessment Scale-Cognitive Subscale (ADAS-Cog), depression (depr), frontotemporal dementia (FTD), asymptomatic mutation carrier (aMC), healthy control (HC), Lewy Body dementia (DLB), mild cognitive impairment (MCI), mini-mental state examination (MMSE), not applicable (NA), Parkinson’s disease (PD), symptomatic mutation carrier (sMC), vascular dementia (VD).

For the three classical complement activator proteins C1q, C1r and C1s, strong evidence for altered levels is lacking, with a trend for increased levels in AD plasma in C1q and C1r (39, 53) while no differences are reported related to C1s (54). Analyses on the plasma concentrations of the alternative complement pathway component fB or its cleaved form Bb also consistently found no differences (39, 53, 54). Similarly, no significant differences were observed in the terminal pathway proteins (39, 54) in AD patients compared to HC. To our knowledge, no evidence for the involvement or increased levels of lectin pathway components in AD has been published. Presumably, it seems that the focal point of changes in AD plasma complement levels is the central axis of the complement system, and not the activator and terminal pathways. Indeed, several studies reported a significant increase in C3 levels in the plasma of AD patients (53, 55–58). Notably, one study reported elevated plasma C3 levels in MCI patients compared to HC, but also compared to AD patients (59). Together, these studies indicate that plasma C3 levels are increased in AD, with a more pronounced increase in the earlier MCI phase. Surprisingly, a meta-analysis by Krance et al. did not endorse these results, underlying the heterogeneity in plasma measurements (39). Substantial variability was indeed detected in the plasma meta-analysis, but the source of the C3 heterogeneity could not be identified. Moreover, a larger scale population study indicated that low baseline levels of plasma C3 are associated with higher AD risk, which was amplified in APOE ϵ44 highly susceptible individuals (60). Case-control comparisons on plasma C4 levels are less consistent. Increased C4 concentrations in AD patients have been reported compared to HC (56, 58, 59, 61). In contrast, other studies did not replicate the increased C4 levels (39, 53, 54). Remarkably, a recent study detected elevated C4 in the saliva of AD patients, albeit without diagnostic utility (68).

For the complement regulators C1 inhibitor, CR1, fH, factor I (fI) and clusterin, case-control studies also indicated changes in their plasma levels. Firstly, one study indicated lower C1 inhibitor levels in AD plasma (53) and another group showed the same for CR1 (59). Interestingly, polymorphisms in the CR1 gene are a well-known AD risk factor replicated in multiple datasets (25–27, 29, 69). Secondly, studies measuring fH levels are divided between unaltered (39, 53, 54, 70) and increased (56, 71) concentrations in the plasma of AD patients compared to HC. Two studies also reported significantly higher fH in MCI subjects *versus* HC (57, 59) while no difference was observed between MCI patients converting to AD compared to non-convertors (54), arguing against the use of plasma fH as a prognostic AD biomarker. Next, reports on the C3b/C4b protease fI are contradicting, with a study showing unchanged levels between AD and HC (54), another study reporting increased levels in AD plasma compared to MCI (59) and a comparison between MCI convertors and non-convertors indicating a decreased fI plasma concentration (54). Finally, levels of the terminal pathway inhibitor clusterin have been shown to be increased in AD (54, 56) and MCI (50) patients, although this observation has been refuted in other reports (39, 48, 50, 63). Of note, AD-related alterations in clusterin levels could be age-dependent, as it has been shown that higher concentrations are associated with increased dementia risk among elderly persons, as opposed to a decreased risk younger elderly people (72).

Despite the overarching trend of altered complement plasma levels in AD, especially in central axis complement proteins and complement regulators, most studies agree regarding their limited suitability as diagnostic biomarkers. For instance, Cheng et al. reported an accuracy of 64.3% of C4 to distinguishing AD from HC, with 64.3% sensitivity and 64.4% specificity (56). Cutler et al. reported that C1 inhibitor gave a sensitivity and specificity of both 58%, and a sensitivity of only 30.2% when the specificity was set at 80% (53). In addition, setting specificity at 80% gave a sensitivity value of 40% for fH in a ROC analysis by Hye et al. (71). Based on the complexity of AD, combinations of complement proteins with each other or with other variables might hold more potential. In a model by Hakobyan et al. combining clusterin with co-variables associated with AD, the predicted specificity was 75% at 70% sensitivity (54). In the same study, a model combining clusterin, TCC and fI with APOE status was predictive of MCI conversion with an AUC of 0.85 (79% predicted specificity at 80% sensitivity). Furthermore, a model from Wang et al. containing C3 and fH together with four other protein markers (ApoeE1, ApoCIII, ApoE, A2 macroglobulin) and age, sex, genotype, and education level covariates could differentiate MCI and AD from HC with an AUC of 0.743 and 0.837 respectively (41). Finally, a model combining fB and fH with age could moderately predict MCI progression to AD (AUC of 0.71) (59).

Similar to CSF, plasma complement levels have also been analyzed as predictor for disease severity. Thambisetty et al. observed a trend (p-value 0.07) for association between plasma C3 and MMSE score in a combined group of AD and MCI subjects (55). Moreover, another study showed the same association for fH, with increased plasma concentrations correlating with increased cognitive decline (decreased MMSE scores) (71). However, the authors made use of semiquantitative immunoblotting, so a validation study with alternative quantitative assays is necessary. The complement-associated protein clusterin is most associated with AD severity. Importantly, clusterin is a multifunctional protein, so this association could also stem from other mechanism than inhibition of the terminal complement pathway. For example, clusterin is also involved in Aβ aggregation and clearance, cholesterol and lipid regulation, and apoptosis (73, 85). Nevertheless, it is recognized as a robust marker of disease severity in both MCI and AD, as its association with cognitive decline (50, 63, 65, 66) and atrophy (50, 66, 67) has been replicated across studies in using different assay platforms. Finally, one study explored the use of complement levels as potential marker for drug efficacy, in which the plasma levels of C1 inhibitor and fH were negatively correlated with the efficacy of the PPARγ agonist rosiglitazone in AD patients (62).

### 3.2 Free complement as biomarker in MS

MS is an inflammatory, autoimmune-mediated disease of the CNS which is characterized by the formation of inflammatory demyelinating lesions and neurodegeneration (86, 87). The exact etiological basis for this disease is still unknown, although it is clear that both genetics and environmental triggers are involved. Nowadays, magnetic resonance imaging (MRI) is still the most important tool for MS diagnosis, disease activity and treatment response (88, 89). In general, at least two demyelinating lesions disseminated in space and time must be identified for diagnosing MS. Additionally, identification of oligoclonal bands and IgG into the CSF of patients is commonly used but is very labor intensive and costly (88, 89).

Also in MS pathology, several reports indicate the link with the complement system. For example, many complement proteins are detected in both white and grey matter lesions (13–16). More specifically, immunohistochemical analysis of MS plaques of patients with progressive MS revealed the presence of several complement proteins (C3, fB, C1q), activation products (C3b, iC3b, C4d, TCC) as well as regulators (fH, C1 inhibitor, clusterin) (13). Regarding the cellular source, reactive astrocytes and microglia are often proposed as primary sources of complement (13, 14). Complement-mediated demyelination is believed to be dominated by the classical pathway, as C1q is highly present in MS plaques and C3 as well as MAC activation is observed in white matter lesions (3, 13). Next to its role in demyelination, complement has been suggested to be implicated in mediating synaptic alterations by being involved in aberrant synaptic pruning. Mainly based on animal studies, C3-mediated synaptic loss is claimed to be linked with microglial activation and phagocytosis (74–76, 90). Finally, several complement KO mouse models illustrate the involvement of complements in MS pathology. For example, a study with C3 KO mice indicates the requirement of C3 for development of maximal experimental autoimmune encephalomyelitis (EAE) disease (77). Also, inhibition of the alternative complement pathway *via* monoclonal antibody-mediated fB inhibition, attenuated chronic EAE but did not prevent disease development (78).

Over the past decades, several studies explored the presence of complement components in the blood and CSF to evaluate their potential as biomarkers for MS diagnosis, disease severity and prognosis. In these studies, the levels of a broad variety of complement components were measured in biofluids from MS patients and compared with HC or non-inflammatory other neurological disease patients (NI-ONDC), or comparing different MS patient subgroups (**Tables 3**, **4**). In the following sections, we describe the overall alterations in complement levels in CSF and blood and discuss which correlations could be established between complement levels and measures for MS severity, progression, patient stratification and treatment response.

**Table 3:** Overview and biomarker potential of complement components evaluated in the cerebrospinal fluid (CSF) of multiple sclerosis (MS) patients compared to a control group.

Cerebrospinal fluid					
Protein	Change	Comparison	Ref.	Biomarker potential	Ref.
C1q	↑	MS (RR, CIS) *vs* HC rMS *vs* HC MS (RR, SP) *vs* NI-ONDC	(79, 91)	Positive correlation with neuroinflammatory and neurodegenerative marker levels	(79)
C1s	(↑)	MS (RR, SP, PP) *vs* HC	(92)	NA	
C1 inhibitor	(↑)	MS (RR, SP, PP) *vs* NIDC	(93)	NA	
C3	↑	MS (NS) *vs* HC rMS *vs* HC MS (PP, SP, RR) *vs* NI-ONDC	(79, 83, 94, 95)	Positive correlation with EDSS score ↑ associated with MRI lesion number (≥9), nerve injury (NFL)	(95)
	↓	RRMS *vs* controls (with low back pain, no white matter abnormalities)	(96)		
	=	MS (NS) *vs* HC MS (NS) *vs* ONDC pMS *vs* controls (NS) CIS *vs* RRMS	(82, 97, 98)		
C3a	↑	MS (RR, CIS) with new T2 lesions during follow-up (4y) vs absence of new T2 lesions EDA-3 vs NEDA-3 scored MS patients (RR, CIS) follow up (1y)	(79)	Positive correlation with neuroinflammatory and neurodegenerative marker levels, T2 and GAD+ MRI lesions at baseline and during follow up.	(79)
	=	MS (RR, CIS) *vs* HC rMS *vs* ONDC	(79, 99)		
iC3b	↑	CIS *vs* NIDC	(93)	NA	
C4	(↑)	MS (RR, SP, PP) *vs* NIDC	(93)	NA	
	↓	pMS *vs* controls (NS)	(98)		
	=	MS (NS) *vs* HC MS (NS) *vs* ONDC MS (RR, SP) *vs* controls (low back pain, no white matter abnormalities) CIS *vs* RRMS	(82, 83, 96, 97)		
C4a	↑	MS (RR, SP, PP) *vs* HC	(80)	NA	
	=	rMS *vs* ONDC	(99)		
C4b	↑	active RRMS *vs* NI-ONDC	(94)	NA	
C5a	=	MS *vs* ONDC	(99)	NA	
TCC (C5b-9)	↑	MS (RR, NS) *vs* NI-ONDC RRMS *vs* ON-MS with ON CIS *vs* NIDC	(93, 100, 101)	Positive correlation with EDSS	(101)
	(↑)	MS (RR, SP, PP) *vs* HC	(92, 102)		
	=	MS (RR, CIS) *vs* HC	(79)		
C9	↓	rMS/pMS *vs* ONDC	(103, 104)	NA	
	(↓)	MS (RR, SP, PP) *vs*. HC	(92)		
	=	MS (NS) *vs* ONDC	(105, 106)		
Factor H	↓	MS *vs* HC active RRMS *vs* NI-ONDC	(94, 107)	NA	
	(↑)	MS (RRMS, pMS) *vs* ONDC	(108)		
Factor B	↓	MS *vs* HC	(107)	NA	
	(↓)	MS (RR, SP, PP) *vs* HC	(92)		
Factor I	↑	CIS *vs* NIDC	(93)	NA	
	=	MS (RR, SP, PP) *vs* HC	(92)		
MASP-2	=	MS (SP, PP) *vs* controls (NS, non-MS)	(81)	NA	
FHR125	↑	CIS *vs* NIDC	(93)	NA	
sCR2	↑	MS (SP, RR) *vs* NI-ONDC	(91)	Positive correlation with MSSS	(91)
Clusterin	(↑)	MS (RR, SP, PP) *vs* HC	(92)	NA	

The characteristics of the patient groups that were compared are indicated in the ‘comparison’ column. The ‘change’ column represents the observed level alteration for the respective complement component between patient populations as represented in the ‘comparison’ column. The ‘biomarker potential’ column includes the studies that indicate a positive correlation between the level of the respective complement component and a measure as assessment of ongoing disease activity or severity, disease progression, patient stratification and/or response to treatment. To cover as much as research as possible, we did not set restrictions concerning the publication date of the references that were retrieved for this review. Symbols: ↑: significant increase; (↑): increased, unsignificant trend; ↓: significant decrease; =: unchanged. clinically isolated syndrome (CIS), evidence of disease activity (EDA), expanded disability status scale (EDSS), Factor H-related (FHR), gadolinium positive (GAD+), healthy controls (HC), magnetic resonance imaging (MRI), mannose-binding lectin-associated serine protease (MASP-2), multiple sclerosis (MS), MS severity score (MSSS), neurofilament light chain (NFL), no evidence of disease activity (NEDA), non-inflammatory other neurological disease controls (NI-ONDC), not applicable (NA), not specified (NS), optic neuritis (ON), primary progressive (PP), relapsing-remitting (RR), relapsing-remitting multiple sclerosis (RRMS), relapsing MS (rMS), secondary progressive (SP), progressive MS (pMS), terminal complement complex (TCC).

**Table 4:** Overview and biomarker potential of complement components evaluated in plasma or serum of multiple sclerosis (MS) patients compared to a control group.

Plasma/serum					
Protein	Change	Comparison	Ref.	Biomarker potential	Ref.
C1s	↑	NMO *vs* RRMS	(109)	NA	
	↓	RRMS *vs* HC	(109)		
	=	MS (RR, SP, PP) *vs* HC	(92)		
C1 inhibitor	↑	MS (RR, SP, PP) *vs* HC NMO *vs* RRMS	(92, 109)	↑ 3 months IFNA treatment (RRMS) *vs* baseline	(110)
	↓	RRMS *vs* HC	(109)		
C3	↑	MS (RR, SP, PP) *vs* HC MS (RR, CIS) *vs* HC rMS *vs* HC	(79, 92)	Positive correlation with EDSS	(111)
	↓	MS (RR, SP) *vs* controls (low back pain, no white matter abnormalities) NMO *vs* RRMS	(96, 109)		
	=	pMS *vs* controls (NS)	(98)		
iC3b	↓	RRMS *vs* HC	(109)	NA	
C3a	↑	MS (RR, PP, SP) *vs* HC	(112)	NA	
	=	MS (RRMS, CIS) *vs* HC early relapse onset MS *vs*(NI-) ONDC rMS vs ONDC	(79, 99, 113)		
C3bc	NA	NA	NA	↑ 3 months IFNA treatment (RRMS) *vs* baseline	(114)
C4	↑	MS (RR, SP, PP) *vs* HC RRMS *vs* HC relapse RRMS *vs* remission RRMS NMO *vs* MS	(92, 111, 115)	Positive correlation with EDSS	(111)
	(↑)	MS (RR, SP, PP) *vs* HC	(80)		
	=	MS (RR, SP) *vs* controls (low back pain, no white matter abnormalities)	(96)		
C4a	↑	MS (RR, SP, PP) *vs* HC Acute/active RRMS *vs* HC pMS vs controls (NS) active RRMS *vs* stable RRMS	(80, 92, 98)	NA	
	↓	MS (RR, SP, PP) *vs* HC NMO *vs* MS (RR, SP, PP)	(112)		
	=	rMS *vs* ONDC early relapse onset MS *vs*(NI-) ONDC	(99, 113)		
C4d	↑	NMO *vs* RRMS	(109)	NA	
C5	↑	NMO *vs* RRMS	(109)	NA	
	=	early relapse onset MS *vs*(NI-) ONDC	(113)		
C5a	↑	NMO *vs* RRMS	(109)	NA	
	=	rMS *vs* ONDC	(99)		
TCC (MAC or C5b-9)	↑	MS (RR, SP, PP) *vs* HC NMO *vs* RRMS	(109, 112)	↑ three and six months IFNA treatment (RRMS) vs baseline	(114)
	↓	RRMS *vs*. HC	(109)		
	=	early relapse onset MS *vs*(NI-) ONDC MS (RRMS, CIS) *vs* HC	(79, 113)		
C9	↑	acute RRMS *vs* stable RRMS	(92)	NA	
	↓	MS (RR, SP, PP) *vs* HC	(92)		
	=	pMS/rMS *vs* ONDC	(103)		
Factor H	↑	MS (RR, SP, PP) *vs* HC Acute/relapse RRMS *vs* stable/remission RRMS pMS (PPMS, SPMS) *vs* RRMS transition rMS to pMS NMO *vs* RRMS	(92, 108, 109, 116)	Positive correlation with EDSS Distinguishing SPMS from RRMS (89.41%, distinction, 69.47% specificity positive predictive value of 72.38%, test cut-off value 4237 mg/l)	(108)
	↓	remission RRMS patients on *vs* off treatment	(108)		
	=	early relapse onset MS *vs*(NI-) ONDC	(113)		
Factor B	=	MS (RR, SP, PP) *vs* HC acute RRMS *vs* stable RRMS	(92)	NA	
Factor Ba	=	early relapse onset MS *vs*(NI-) ONDC	(113)	NA	
Factor Bb	↑	NMO *vs* RRMS	(109)	NA	
	↓	RRMS *vs* HC	(109)		
	=	MS (RR, SP, PP) *vs* HC	(92)		
Factor I	=	MS (RR, SP, PP) *vs* HC early relapse onset MS *vs*(NI-) ONDC	(92, 113)	NA	
MASP-2	↑	MS (SP, PP) *vs* controls (NS, non-MS)	(81)	NA	
Clusterin	=	MS (RR, SP, PP) *vs* HC	(92)	NA	

The characteristics of the patient groups that were compared are indicated in the ‘comparison’ column. The ‘change’ column represents the observed level alteration for the respective complement component between patient populations as represented in the ‘comparison’ column. The ‘biomarker potential’ column includes the studies that indicate a positive correlation between the level of the respective complement component and a measure as assessment of ongoing disease activity or severity, disease progression, patient stratification and/or response to treatment. To cover as much as research as possible, we did not set restrictions concerning the publication date of the references that were retrieved for this review. Symbols: ↑: significant increase; (↑): increased, unsignificant trend; ↓: significant decrease; =: unchanged. clinically isolated syndrome (CIS), expanded disability status scale (EDSS), healthy controls (HC), Glatiramer acetate (GA), interferon alpha (IFNA), mannose-binding lectin-associated serine protease (MASP-2), MS severity score (MSSS), multiple sclerosis (MS), neuromyelitis optica (NMO), non-inflammatory other neurological disease controls (NI-ONDC), not applicable (NA), not specified (NS), optic neuritis (ON), progressive MS (pMS), primary progressive (PP), secondary progressive (SP), relapsing MS (rMS), relapsing-remitting (RR), relapsing-remitting multiple sclerosis (RRMS), Response Gene To Complement 32 (RGC-32), terminal complement complex (TCC).

#### 3.2.1 Complement proteins levels in CSF and their biomarker potential

CSF is a biofluid that is frequently collected by lumbar punction as part of the MS diagnosis process to test oligoclonal banding as a sign of intrathecal antibody synthesis (86). **Table 3** represents an overview of complement components that were examined in the CSF of MS patients compared to a control group. In general, the alterations of complement components in the CSF of MS patients compared to a certain control group are quite cohesive between studies. Most studies indicate that the amount of complement factors is significantly increased (C1q, C4a) or unchanged (C3a, C4, MASP-2, fI) in the CSF of MS patients compared to HC (79–83, 92). Exceptions are fH and fB, which showed a significant decrease in CSF of MS patients compared to HC (94, 107). For complement C3, research outcomes are conflicting, as two studies indicate a significant increase (79, 83) while one study did not observe a difference in CSF C3 levels (82) in MS patients compared to HC. Only two studies evaluated CSF TCC levels in MS patients *versus* HC (79, 102). Mollnes et al. reported increased TCC levels in 30% of the MS patients (102), while Håkansson et al. reported that there was no difference in TCC levels between MS patients and HC (79). Instead of HC, a lot of studies included (NI-)ONDC patients as control group. Here, the majority of investigated complement factors was elevated (C1q, C1 inhibitor, iC3b, C4b, fI, fH, TCC, FHR125, soluble Complement Receptor 2 (sCR2)) or unchanged (C3a, C4, C4a, C5a) in CSF of MS patients compared to NI-ONDC patients (82, 91, 93–96, 99–101, 108). Of the examined complement factors, fH was unanimously decreased (94, 103, 104). For C3, two studies reported a significantly increased amount in MS patients *versus* NI-ONDC (94, 95). In contrast, one study could not detect a difference in C3 levels (82) and another study described decreased C3 levels in MS patient compared to a control group with low back pain (without white matter abnormalities) (96). The same was true for terminal factor C9, for which two studies indicated a significant decrease in MS *versus* ONDC (103, 104), while two other studies could not detect differences between MS and ONDC (105, 106). Also for C4, CSF levels were increased in one study (93) while unchanged in another study (82). A few studies also investigated whether CSF complement levels were different between specific subgroups of MS patients. For example, comparing clinically isolated syndrome (CIS) patients with relapsing-remitting (RR) MS patients did not show any changes in C3 or C4 levels (97). Interestingly, C3a levels in the CSF of RRMS and CIS patients were significantly elevated when they encountered new T2 MRI lesions during a follow-up period of 4 years compared to RRMS and CIS patients that did not develop new MRI lesions during the same follow-up period (79). Moreover, C3a levels were also significantly increased in patients that received evidence of disease activity (EDA)-3 scoring compared to no evidence of disease activity (NEDA)-3 scoring patients during a follow-up time of one year (79).

Although investigated by a limited number of studies, some correlations were discovered between the level of a single complement factor and a measure for ongoing disease activity or severity, disease progression and patient stratification (**Table 3**). Most of the significant correlations could link a complement level to a measure for disease severity or progression. For example, a positive correlation was observed between CSF C3 levels and expanded disability status scale (EDSS) score (95), CSF TCC levels and EDSS score (101) and CSF sCR2 levels and MS severity score (MSSS) score (91). Furthermore, C1q levels and C3a levels were both positively correlated with the level of neuroinflammatory and neurodegenerative markers in the CSF (79). Remarkably, C3a levels were positively correlated with the amount of gadolinium-positive and T2 MRI lesions present at baseline and after follow-up (79).

Some studies also aimed to determine the source of complement factors in the CSF by measuring CSF:blood ratios of certain complement factors. An increased CSF:blood ratio suggests that elevated CSF complement levels are either a consequence of peripheral leakage due to dysfunctional brain barrier function or intrathecal complement synthesis. To clarify this, researchers studied whether a correlation exists between an increased CSF:blood ratio for complement proteins and an increased CSF:blood ratio for albumin and/or a raised IgG index, which are considered as two measures for MS disease activity (117–119). One study recorded significantly increased CSF:serum ratios for fH in MS patients compared to ONDC, which was strongly correlated with CSF:serum albumin ratio (Pearson’s correlation = 0.83, *P* < 0.001), suggesting influx of fH from periphery to CSF due to brain barrier dysfunction (108). Also for complement proteins C1s and clusterin, the CSF:plasma ratio correlated with CSF:serum albumin ratio (92). On the other hand, CSF:serum ratio for C4a was increased in MS patients compared to HC, but CSF C4a levels showed moderate correlation with CSF IgG (*r* = 0.53, *p* = 0.01) while not with CSF albumin, suggesting C4a CSF was more likely the result of intrathecal synthesis (80). For C9 and fB, CSF:plasma ratio correlated with both CSF:serum albumin ratio and IgG index (92).

#### 3.2.2 Complement protein levels in the blood and their biomarker potential

Over the years, a broad variety of complement components present in the blood was studied in MS patients *versus* different control groups. As discussed above, blood is a more accessible biofluid than CSF, but it is also more prone to non-disease specific influences such as general inflammation. Therefore, it is not surprising that studies regarding complement level alterations in the blood show more variable results compared to CSF analyses. An overview of complement components that were examined in the blood of MS patients compared to controls is shown in **Table 4**.

In the blood, several studies reported upregulated C3, C4 and fH levels in MS patients compared to HC (79, 80, 92, 108, 112, 115, 116). However, in case of C3 this increase rendered insignificant when an adjustment for high-sensitivity C-reactive protein (hsCRP) levels was performed *via* covariance analysis, meaning that C3 levels in the plasma were affected by hsCRP as a result of systemic inflammation rather than MS disease itself (79). For fB, fI and clusterin, no difference in blood levels between MS patients and HC could be detected (79, 92), while iC3b and C9 were described to be decreased in MS compared to HC (92, 109). Several studies also reported alterations in MS *versus* HC for C1s, C1 inhibitor, C3a, C4a, TCC and factor Bb but the results of these studies were conflicting. Blood levels of C1 inhibitor and C4a were increased (80, 92) or decreased (109, 112) in MS *versus* HC. C1s and factor Bb levels were shown to stay unchanged (92) or decreased (109). In contrast, C3a was reported to stay unchanged (79) or increased (112). Also for TCC, the results of the different studies are variable, as it was reported to be increased (112), decreased (109) or unchanged (79) when MS was compared with HC. Most studies that implemented (NI-)ONDC patients as control group concluded there was no difference with the blood levels of the investigated complement factor (*i.e.*, C3a, C4, C4a, C5, C5a, C9, fH, factor Ba) compared to (NI-)ONDC patients (96, 99, 103, 113), except one study which reported decreased C3 levels in serum of MS patients *versus* control patients with low back pain (96). Some complement factors were also found to differ between certain MS patients groups or between MS patients and other demyelinating diseases such as neuromyelitis optica (NMO). For example, increased levels of C1s, C1 inhibitor, C4d, C5, C5a, fH, factor Bb and TCC and decreased levels of C3 were detected in the blood of NMO patients *versus* MS patients (109). Within MS patient populations, acute or active RRMS patients showed increased levels of C4a, C9 and fH compared to stable RRMS patients (80, 92, 108).

Regarding the biomarker potential of blood detected complement components, a positive correlation between the level of C3, C4 and fH and EDSS score as a measure for disease activity or severity was described (108, 111). Furthermore, fH also positively correlated with MSSS scores and was a valuable marker for distinguishing secondary progressive (SP) MS from RRMS, with a positive prediction value of 72,4%. fH is therefore an interesting biomarker candidate as indicator of disease progression and disease course (108). To our knowledge, no study found a real correlation between complement levels and response to therapy. However, it was reported that after three months interferon alpha (IFNA) treatment, C1 inhibitor and TCC levels were elevated in RRMS patients compared to their baseline levels (110, 114). For TCC, blood levels were also increased six months of IFNA treatment compared to baseline levels (114). Instead of complement factors themselves, the expression of a gene induced by complement activation, namely Responsive Gene to Complement 32 (RGC-32), could have remarkable biomarker potential for the prediction of both relapse and responsiveness to Glatiramer acetate (GA) therapy (120). Kruszewski et al. investigated the expression level of RGC-32 in peripheral blood mononuclear cells and could show decreased expression of RGC-32 in peripheral blood mononuclear cells (PBMCs) of RRMS patients in acute disease state compared to RRMS patients in remission (120). On the contrary, GA responders showed upregulation of RGC-32 expression compared to GA non-responders (120). By implementing ROC analysis, they could claim that RCG-32 expression levels could predict the probability for relapse and GA responsiveness with 90% and 85% probability, respectively (120).

#### 3.2.3 Biomarker potential of combined complement factors in MS

As summarized in **Tables 3**, **4**, few correlations are described between the level of one single complement component and a measure for ongoing disease activity or severity, disease progression, patient stratification and/or response to treatment. However, the combination of multiple complement factor could be a stronger tool to create predictive models. Indeed, a few studies reported logistic regression (LR) models encompassing a set of complement factors together with other clinical patient characteristics to be valuable as a predictive tool. Combining C9 plasma levels, disease duration and age resulted in a LR model to predict for clinical relapse within a group of RRMS patients (AUC 0.73) (92). In the same research, another LR model combining C3, C9, C1 inhibitor and fH plasma levels could predict the probability of MS compared to HC (AUC 0.97) (92). Another study created a LR model comprising plasma levels of C1 inhibitor and TCC, which was effective to distinguish NMO from MS patients (AUC 0.98) (109). Furthermore, a model which combined CSF levels of C3, C9, fB, C1q, fI and properdin with patient age was also valuable for distinguishing NMO from MS (AUC 0.81) (93). In a study with relatively small patient groups, the CSF levels of C3, C4, IgM, mononuclear cells, neuron-specific enolase, S100 and lactate, as well as CSF:blood albumin ratio and IgM index were taken into account to calculate a score that could successfully discriminate between RRMS and SPMS patients (121).

### 3.3 Free complement as biomarker in other neurological diseases

The involvement of the complement system in the pathology of other neurodegenerative and neuroinflammatory diseases such as Parkinson’s disease (122), ischemic stroke (122, 123), glioblastoma multiforme (124), is extensively reviewed by others. Compared to AD and MS, the applicability of free complement as biomarker was less extensively studied in these other pathologies. Therefore, the biomarker potential of free complement in other neurological diseases will not be addressed in this review.

## 4 The biomarker potential of complement-containing extracellular vesicles

As discussed previously, complement proteins are involved in the pathology of several neuroinflammatory and neurodegenerative diseases. Even though the level of free complement proteins in both blood and CSF has been thoroughly studied (**Tables 1** **–** **4**), there is no clear consensus for implementation of complement proteins as biomarkers until today. However, the consideration of shifting to complement-containing extracellular vesicles (EVs) as interesting biomarkers instead of free complement proteins has gained progressive attention during the past five years.

### 4.1 Extracellular vesicles

EVs are nanosized double membrane particles which are released by a broad variety of cell types (125, 126). They carry a lot of biological information (*i.e.*, proteins, nucleic acids, metabolites, lipids) which typically resembles the state of their cell of origin. This biological information packed within EVs can be transferred to other nearby or distant cells, which categorizes EVs as an important form of intercellular communication (127). Dependent on their way of biogenesis, EVs can be further divided into subclasses. Exosomes are formed as intraluminal vesicles (ILVs) within multivesicular bodies as a part of the endosomal pathway (127). Subsequently, MVBs fuse with the plasma membrane to release the ILVs as exosomes (127). The second EV subtype are ectosomes or microvesicles, which are formed by direct outwards budding of the plasma membrane (127, 128). A third group of EVs, the apoptotic bodies, are specifically formed during apoptotic cell death *via* random blebbing of the plasma membrane (127). Despite the description of these different EV subtypes, discriminatory subtype-specific markers are lacking (125, 129). For this reason, we will collectively use the term EV in this review.

EV research, especially in the context of biomarker studies, needs to fulfill the requirements for high standardized isolation and quality control. Therefore, the Minimal Information for Studies of EVs (MISEV) guidelines were developed and are continuously updated (129). EV sample preparation procedures as well as EV source information must be accurately described. This includes a description of the volume of fluid, and/or cell number, and/or tissue mass from which EVs were extracted, as well as the quantification of EV amount per volume of initial fluid or per number of producing cells/mass of tissue by implementing two distinct methods such as assessing protein amount, particle number and lipid amount. Additionally, EV nature are recommended to be verified by checking the presence of at least three protein markers. More specifically, analysis of at least one transmembrane or glycosylphosphatidylinositol (GPI)-anchored protein associated with the plasma membrane and/or endosomes (general or cell-/tissue-specific), one cytosolic or periplasmic protein marker and one non-EV co-isolated structure is required to prove the presence and purity of the EV preparations. Additionally, researchers can only claim the nature of small EVs by evaluating an extra set of markers, which comprises proteins that are situated in/on intracellular cellular compartments, including the Golgi apparatus, mitochondria, autophagosomes, peroxisomes and the endoplasmic reticulum. Proteins associated with these intracellular compartments are normally not enriched in smaller EVs (<200 nm diameter) (129).

### 4.2 Why EVs are interesting in the context of biomarker studies

The consideration of shifting from free protein levels to EV-associated protein levels in biomarker research stems from the fact that EVs have multiple characteristics regarding biomarker potential, which could overcome some of the limitations of free proteins. Firstly, the membranous nature of EVs makes them stable carriers that can protect their cargo from degradation (130). Secondly, it is possible to unravel the cellular source of EVs *via* analyzing the presence of cell-specific markers (131). In the context of disease, it can be an advantage to focus on EVs carrying the protein of interest originating from a cell type that is specifically engaged in the pathology to narrow down off-target, non-specific sources. For CNS diseases, it is a major advantage that brain-derived EVs can cross brain barriers and can thereby be isolated from peripheral biofluids (132). These peripheral liquid biopsies are more easy to collect compared to CSF and are a less expensive alternative to imaging (132). For example, astrocyte EVs are often defined by the presence of L-Glutamate/L-Aspartate Transporter (GLAST), while L1 cell adhesion molecule (L1CAM) is an extensively studied marker to enrich for neuronal EVs (131, 132). Thirdly, EVs carry disease specific signatures as they mimic the status of their cell of origin (130, 132). Knowledge about cellular EV origin can not only be an advantage for diagnostic purposes, but also allows to gain more insights into the underlying disease mechanisms itself (130, 133, 134). Finally, analysis of EVs can improve measurement sensitivity and signal-to-noise ratio, specifically when enriched for a certain cell type-specific EV population (132, 135). The improved sensitivity can be illustrated by the fact that alterations in plasma EVs are often absent in complete plasma (132). Since most EVs present in the blood do not originate from the CNS, the choice to enrich for EVs produced by neurons or glial cells can improve signal-to-noise ratio (132).

In the following sections, we will describe the findings of studies on the complement content of EVs in different neurological pathologies, which illustrates their growing importance in the field of biomarker identification. The most important conclusions regarding EV-associated complement, as well as EV characterization and EV quality control conducted by the studies discussed in this review are summarized in **Table 5**.

**Table 5:** Minimal Information for Studies of EVs (MISEV) guidelines related information about EV isolation and characterization in the studies discussed in this review, investigating human- and mouse-derived EVs.

Patient studies									
Patient info (disease/controls)			Biofluid/tissue as EV source	EV cellular source (marker)	Complement content EVs	EV isolation	EV characterization		Ref.
							Global quantification	Protein marker detection	
Dementia	FTLD (genetic GRN/C9orf72, sporadic)	HC	Plasma	Bulk	↑ C1q, C3, C4 cargo per EV in FTLD ↓ C4 cargo in GRN+(homo) *vs* GRN+(het) and sporadic ↑ C4 cargo in sporadic *vs* GRN+(het) ↑ C4 EV/plasma ratio in sporadic *vs* GRN+ FTLD ↓ C4 EV/plasma ratio in GRN+ (homo) *vs* HC and other FTLD groups ↑ C3 EV/plasma ratio in sporadic *vs* HC and other FTLD groups	Total Exosome Isolation kit	☐ A ☒ B (NTA) ☐ C ☐ D ☐ E	☒ 1 (CD9) ☒ 2 (TSG101) ☐ 3 ☒ 4 (Calnexin)	(136)
	AD, FTLD	A/G HC	Plasma	Bulk (CD81+) Astrocytes (GLAST+) Neurons (L1CAM+)	AEVs and NEVs of AD patients are neurotoxic (induction Membrane Attack Complex (MAC) expression, membrane disruption, reduced neurite density, decreased cell viability)	ExoQuick + IP	☒ A (0,5 ml) ☒ B (NTA) ☒ C (Bradford) ☐ D ☒ E	☒ 1 (CD81, CD9, CD63) ☒ 2 (ALIX) ☒ 3 (ApoA1) ☒ 4 (GM130)	(137)
	AD (mild) AD (moderate)	A/G HC Pre-clinical AD	Plasma	Astrocytes (GLAST+)	↑ C1q, C4b, C3d, factor B, factor D, fBb, C3b and TCC (C5b-C9) in AEVs of mild AD *vs* HC. Mean complement levels higher in moderate AD *vs* preclinical AD.	ExoQuick + IP	☒ A (250 µl) ☐ B ☐ C ☐ D ☐ E	☒ 1 (CD81, CD59, CD55) ☐ 2 ☐ 3 ☐ 4	(138)
	MCIC AD (mild, moderate)	MCIS A/G HC	Plasma	Astrocytes (GLAST+)	↑ C1q, C4b, fD, fragment Bb, C5b, C3b, C5b-C9 in AEVs of MCIC *vs* MCIS. ↓ CD46, CD59, and type 1 complement receptor in AEVs in MCIC *vs* MCIS.	ExoQuick + IP	☒ A (250 µl) ☐ B ☐ C ☐ D ☐ E	☒ 1 (CD81, CD59, CD55) ☐ 2 ☐ 3 ☐ 4	(139)
	AD Braak stage V-VI	A/G HC	Brain tissue (parietal cortex)	Microglia (CD11b+)	Proteomic EV analysis ↑ C4, CD59 in MEVs from AD *vs* HC	GentleMACS tissue dissociation Sucrose density gradient UC + IP	☐ A ☒ B (TRPS) ☐ C ☒ D (lipidomics) ☒ E	☒ 1 (CD9, CD81, CD63, CD11b) ☒ 2 (syntenin-1) ☒ 3 ☒ 4 (GM130, Calnexin)	(140)
Parkinson’s disease	PD HY stages II and III	HC	Plasma	Bulk	Proteomic EV analysis ↓ Clusterin, C1r in PD *vs* HC	SEC (EV-Second)	☒ A (200 µl) ☐ B ☒ C (Bradford) ☐ D ☒ E	☒ 1 (CD81, CD9) ☐ 2 ☐ 3 ☐ 4	(141)
	PD Mild and severe	HC	Serum	Bulk	Proteomic EV analysis ↓ C1q in PD *vs* HC ↑ Clusterin, C1r in progression from mild to severe PD ↓ C1q in progression from mild to severe PD	DUC	☒ A (5 ml) ☐ B ☒ C (BCA) ☐ D ☐ E	☐ 1 ☐ 2 ☐ 3 ☐ 4	(142)
Multiple sclerosis	RRMS, pMS	HC	Plasma	Astrocytes (GLAST+)	↑ C1q, C3b/iC3b, C5, C5a, fH (pMS vs. HC) ↑ C1q, C3, C3b/iC3b, C5, C5a, fH (RRMS *vs* HC) No difference: C4, C9, fB (MS vs. HC)	ExoQuick + IP	☒ A (0,5 ml) ☒ B (NTA) ☐ C ☐ D ☐ E	☐ 1 ☐ 2 ☐ 3 ☐ 4	(143)
	RRMS	IIH	CSF	Bulk	Proteomics on CSF-EVs C3b, C4, C6, fB, fH uniquely enriched in RRMS-EVs *vs* RRMS-CSF, while not enriched in IIH.	Precipitation + SEC (ExoSpin)	☒ A (5 ml) ☒ B (NTA) ☒ C (NanoDrop) ☐ D ☒ E	☒ 1 (CD81, CD9) ☒ 2 (TSG101) ☒ 3 (HSA) ☐ 4	(144)
	MS	NA	CSF	NA	MAC-containing “vesicles”	NA	☐ A ☐ B ☐ C ☐ D ☐ E	☐ 1 ☐ 2 ☐ 3 ☐ 4	(145)
Ischemic stroke	Symptomatic IS	HC	Serum	Bulk	Proteomic EV analysis ↑ C1qB, C1r in IS *vs* HC	ExoTrap	☒ A (500 µl) ☐ B ☒ C (BCA) ☐ D ☐ E	☒ 1 (CD9, CD81) ☒ 2 (TSG101) ☐ 3 ☒ 4 (Calnexin)	(146)
	IS (CS, CSC)	HC	Serum	Bulk Neurons (L1CAM+)	Proteomic EV analysis C1qA: unique for serum EVs and NEVs from CSC-IS patients. C3 abundant in IS (CSC and CS) and HC in serum EVs and NEVs.	ExoQuick Ultra (Bulk) SmartSEC + IP (NEVs)	☐ A ☒ B (NTA) ☐ C ☐ D ☒ E	☒ 1 (CD63, CD81) ☒ 2 (ALIX) ☒ 3 (Albumin) ☐ 4	(147)
Brain tumor	GBM	HC	Plasma	Bulk	Proteomic EV analysis ↑ C3, C5, C1q, fH in GBM *vs* HC	DUC	☒ A (5 ml) ☒ B (NTA) ☒ C (MicroBCA) ☐ D ☒ E	☒ 1 (CD81, CD9, CD63, CD41a) ☒ 2 (HSP70) ☐ 3 ☒ 4 (GM130)	(148)
	GBM	HC	Plasma	Bulk	Proteomic EV analysis ↑ C3, C4b in GBM *vs* HC	DUC	☒ A (15 ml blood) ☒ B (NTA) ☐ C ☐ D ☒ E	☒ 1 (CD9, CD63) ☒ 2 (TSG101) ☐ 3 ☐ 4	(149)
	GBM Grade II-IV	MEN HC	Plasma	Bulk	Proteomic EV analysis ↑ C3 in GBM *vs* controls	SEC (qEV, Izon)	☒ A (0.5 ml) ☒ B (NTA) ☐ C ☐ D ☒ E	☒ 1 (CD9, ITGA2B, ITGA6, PDCD6IP) ☒ 2 (ANXA1/2/6, FLOT1, HSP90A1B, GAPDH, HIST1H4A) ☐ 3 ☒ 4	(150)
*Mouse studies/primary culture studies*									
Mouse model Cell culture			Biofluid/tissue as EV source	EV cellular source (marker)	Complement content EVs	EV isolation	EV characterization		Ref.
							Global quantification	Protein marker detection	
Dementia	AβO CPE primary culture	Scram-bled CPE primary culture	Culture medium	CPE cells	Proteomic EV analysis ↑ C3 in AβO-stimulated CPE *vs* scrambled	SEC (qEV)	☒ A (500 µl) ☒ B (NTA) ☐ C ☐ D ☒ E	☒ 1 (CD81, CD9) ☒ 2 (TSG101) ☐ 3 ☒ 4 (Calnexin)	(151)
	2xTg-AD 5xFAD 3xTg-AD	WT	Plasma	Neuron (L1CAM+) Astrocyte (GLAST+)	↑ AEV C1q in 3xTg-AD *vs* WT	IP (ExoSORT)	☐ A ☒ B (NTA) ☒ C (Bradford) ☐ D ☐ E	☒ 1 (CD81, CD9, CD63) ☒ 2 (FLOT1) ☒ 3 (APOA, albumin) ☒ 4 (Calnexin)	(152)
	CAST.APP/PS1	CAST WT HC	Brain tissue	Bulk	Proteomic EV analysis C1qa, C1qc increased in EVs from CAST APP/PS1 *vs* WT, but not significant	DUC + sucrose gradient	☒ A (0.4g) ☒ B (NTA) ☒ C (BCA) ☐ D ☒ E	☒ 1 (CD9, CD81, CD63, ITGA) ☒ 2 (ANXA5) ☐ 3 ☒ 4 (GM130, CYC1)	(153)
Brain tumor	GBM mouse model Longitudinal samplings (baseline, T1, T2)	NA	Serum	Bulk	Proteomic EV analysis Complements among group of deregulated proteins in EVs C1ra and C1sa deregulated in EVs between T2 and T1 stages.	Precipitation (Total Exosome Isolation reagent) or SEC	☒ A (50µl) ☒ B (DLS) ☐ C ☐ D ☒ E	☒ 1 (CD9, ITGA) ☒ 2 (ANXA4, ANXA5, ANXA7) ☐ 3 ☐ 4	(154)
	GBM mouse model Longitudinal samplings (baseline, pre-symptomatic T1, symptomatic T2)	NA	Serum	Bulk	Proteomic EV analysis ↓ C4b in T1 *vs* baseline ↑ C1qa, C1ra, C1s1 in T1 and T2 *vs* baseline	SEC (qEV – 70nm)	☒ A (50µl) ☐ B ☒ C (MicroBCA) ☐ D ☐ E	☒ 1 (ITGB1) ☒ 2 (HSPA8, ACT) ☒ 3 (Albumin) ☐ 4	(155)

For global EV quantification requirements, consult checkboxes A-E. Checkbox A) Cell number/fluid volume/tissue mass from which EVs were isolated. Checkbox B) Analysis of particle number. Checkbox C) Analysis of protein amount. Checkbox D) Analysis of lipid amount. Checkbox E) Analysis by electron microscopy. For information regarding EV protein marker detection that has been conducted by the indicated studies, consult checkboxes 1-4. Checkbox 1) Transmembrane or Glycosylphosphatidylinositol (GPI)-anchored protein(s) localized in cells at plasma membrane or endosomes. Checkbox 2) Cytosolic protein(s) with membrane-binding or -association capacity. Checkbox 3) Assessment of presence/absence of expected contaminants. Checkbox 4) For small EVs <200nm: verifying protein(s) associated with compartments other than plasma membrane or endosomes. extracellular vesicles (EVs), astrocyte-EV (AEV), neuronal-EV (NEV), microglial-EV (MEV), not described (ND), not applicable (NA), idiopathic intracranial hypertension (IIH); multiple sclerosis (MS), relapsing-remitting MS, progressive MS (pMS), Alzheimer’s disease (AD), Fronto-Temporal Lobar Degeneration (FTLD), Parkinson’s disease (PD), Glioblastoma multiforme (GBM), meningioma (MEN), ischemic stroke (IS), subcortical (SC), cortical-subcortical (CSC), mild cognitive impairment (MCI), MCI stable (MCIS), MCI converting to dementia (MCIC), Hoehn and Yahr (HY), healthy controls (HC), age-gender matched healthy controls (A/G HC), homozygous (hom), heterozygous (het), Nanoparticle Tracking Analysis (NTA), Bicinchoninic Acid Assay (BCA) Bradford assay (BA), mass spectrometry (MS), differential ultracentrifugation (DUC), ultracentrifugation (UC), Annexin A5 (ANXA5), flow cytometry (FC), transmission electron microscopy (TEM), size exclusion chromatography (SEC), high sensitivity flow cytometry (hsFC), immunoprecipitation (IP), Tunable Resistive Pulse Sensing (TRPS), factor H (fH), factor B (fB), factor D (fD), terminal complement complex (TCC), human serum albumin (HSA), heat-shock protein (HSP), A-beta oligomers (AβO), choroid plexus epithelial cells (CPE), wildtype (WT).

### 4.3 Complement-containing EVs in dementia

Among the different CNS diseases, complement-associated EVs have been investigated most intensively in the context of dementia, more particularly in AD. Moreover, most of the AD-focused studies aim to specifically investigate astrocyte-derived EVs (AEVs), because astrocytes play an important role during AD pathology. While astrocytes have highly important neuronal supportive functions during homeostatic conditions (156, 157), they transform to a proinflammatory (A1) phenotype during neurodegenerative diseases, mediating neurotoxicity *via* a mechanism that is currently not completely elucidated (158–160). Importantly, pro-inflammatory A1 astrocytes are shown to highly upregulate the expression of complement protein C3 in brain tissue samples of AD patients and AD mouse models (161–163). The involvement of astrocyte-related complement upregulation in relation to the astrocyte-mediated neurotoxicity has been proposed, but the question remains whether astrocytes are the main complement source in AD (37, 159, 161).

Analysis of complement content of plasma-derived AEVs from AD patients *versus* age- and gender- matched HC indicated significantly increased factors of the classical and alternative pathways (C1q, C4b, C3d, C3b, fB, fD, fBb, and TCC), while mannose-binding lectin levels were unchanged (138, 139). A similar set of increased complement components (C1q, C4b, fD, fBb, C5b, C3b, and TCC) was observed in patients with MCI that converted to dementia within 3 years (MCIC) compared to stable MCI patients (MCIS) (139). The observation that the mean complement levels in AEVs were higher in patients with moderate AD compared to preclinical AD was further confirmed in a longitudinal study where AD patients were tracked over a period of 5 to 12 years (138). Furthermore, AEV levels of complement regulatory proteins (CD46, CD59, CR1, decay accelerating factor) were decreased in AD *versus* HC (138) and MCIC *versus* MCIS (139). On top of the complement enrichment, these AEVs were also characterized by an elevated inflammatory content as their IL-6, IL-1β and TNF loading was higher in AD patients compared to HC (138). Remarkably, when comparing complement levels in AEVs and neuron-derived EVs (NEVs), the level of investigated complement components in NEVs is 6- to 50- fold lower than in AEVs (138). These findings therefore support the hypothesis that these complement-enriched, inflammatory AEVs are contributing as astrocyte-produced factors that are conceivably neurotoxic during the late inflammatory phase of AD (138). This hypothesis is strengthened by another study that reported the ability of AD patient plasma-derived AEVs to induce MAC deposition on neurons, accompanied with disruption of neuronal membrane integrity, reduction of neurite density and the reduction of neuronal viability (137). On top of this, these results indicate that AEV-associated complement proteins may be implemented as predictive biomarkers for MCI to AD conversion (138, 139). Next to AEVs, microglial-EVs (MEVs) and NEVs are also studied in the context of AD. Regarding microglial-derived EVs (MEVs), one study isolated MEVs from human cortex tissue and reported elevated MEV-associated C4 levels, as well as upregulated complement regulator CD59 in AD *versus* HC (140). In contrast to AD, EV-associated complement in frontotemporal lobar degeneration (FTLD) was only investigated once, in which sporadic as well as genetic forms of FTLD (heterozygous/homozygous GRN mutation carriers, intermediate/pathological C9orf72 expansion carriers) were included. Although the concentration of plasma EVs was lower in sporadic and genetic FTLD patients, the C1q, C3 and C4 cargo per EV was higher in FTLD compared to EVs from HC (136). Additionally, EV-complement related differences between different subgroups of FTLD were also detected. For example, C4 cargo was decreased in GRN+ homozygous genetic FTLD *versus* heterozygous GRN+ and pathological C9orf72 genetic FTLD, while EV-associated C4 increased in sporadic FTLD compared to heterozygous GRN+ FTLD (136). C1q, C3 and C4 EV:plasma ratios were also compared between the different groups. Compared to HC, C3 EV:plasma ratios were elevated in sporadic FTLD while C4 EV:plasma ratios were decreased in homozygous GRN+ FTLD (136). Moreover, C4 EV:plasma ratios were increased in sporadic FTLD *versus* GRN+ genetic FTLD but decreased in homozygous GRN+ FTLD *versus* all other FLTD groups (136). For C1q, no differences in EV:plasma ratios could be detected amongst all groups investigated (136).

Next to patient studies, there is a limited amount of EV research on animal models for AD, in which complement components were found into EVs. C1q levels were shown to be increased in plasma-derived AEVs from 3xTg-AD mice compared to WT mice (152). Moreover, C1q levels in plasma-derived AEV were positively correlated with C1q levels in the hippocampus and cortex, which implies that C1q levels in AEVs can reflect C1 levels in the indicated brain regions (152). Another study showed an insignificant increase in C1qa and C1qc levels in CAST APP/PS1 mice brain derived EVs compared to EVs from WT control mice (153). Moreover, we previously reported an increased release of C3-containing EVs by choroid plexus epithelial (CPE) cells that were stimulated with amyloid-beta oligomers (AβO), identifying a novel source of complement-containing EVs that might potentially be detected in the CSF as biomarker for AD (151).

### 4.4 Complement-containing EVs in Parkinson’s disease

For PD, two small-sized proteomic studies on blood-derived EVs from sporadic PD patients in different progression stages compared to HC revealed alterations in the level of EV-associated complement proteins (141, 142). Both studies aimed to identify potential EV-associated biomarkers for PD diagnosis and PD progression. A pilot study with 16 PD patients, stratified *via* the stage of PD progression according to the Hoehn and Yahr (HY) stages (HY stages II and III), observed a significant decrease of complement proteins clusterin and C1r, as well as apolipoprotein A1 (ApoA1) in plasma EVs from PD patients *versus* HC (141). Therefore, these three EV-enriched proteins may be proposed as potential biomarker candidates for PD diagnosis (141). However, only ApoA1 present in the EV fractions could be correlated with PD progression, as ApoA1-EV levels were decreased in PD patients with HY stage III *versus* HY stage II, while plasma protein levels of ApoA1 remained unchanged (141). However, we want to remark that ApoA1 has been indicated as a contaminant that often co-isolates with EVs during EV preparations according to the MISEV guidelines (129). On the contrary, the second proteomic study on serum EVs, including 20 PD patients subdivided according to the HY scale into mild (HY < 3) and severe (HY > 3), could identify complement proteins that were significantly altered during PD disease progression (142). Here, increased EV levels of clusterin and C1r were detected in PD patients with progression from mild to severe disease, whereas decreased levels of EV-associated C1q were detected in PD patients *versus* HC as well as during progression from mild to severe PD (142). These results indicate a potential for clusterin, C1r and C1q as EV biomarkers for PD progression (142). In conclusion, when comparing both PD studies, both C1r and clusterin might be interesting biomarkers for PD diagnosis and/or progression, but validation with larger patient cohorts is essential.

### 4.5 Complement-containing EVs in MS

The first observation of complement-associated vesicles in MS was made over 30 years ago by Scolding et al., who discovered the presence of MAC-containing vesicles in the CSF of MS patients (145). These MAC-containing vesicles were also found to be produced by oligodendrocytes as a response to complement activation (145). More recently, two patient studies report altered complement EV content in biofluid samples of MS patients. One proteomic analysis of EVs isolated from the CSF of RRMS patients revealed a unique enrichment of several complement proteins (C3b, C4, C6, fB, fH) in EVs compared to CSF levels, while these were not enriched in CSF-EV samples of idiopathic intracranial hypertension control patients (144). We found one study that investigated the biomarker potential of complement-containing EVs in MS. In this study, the potential of circulating NEVs and AEVs as biomarkers for complement-mediated synaptic loss during MS was examined (143). Therefore, synaptic proteins were analyzed in NEVs while a wide spectrum of complement components (C1q, C3, C3b/iC3b, C4, C5, C5a, C9, fB, fH) was measured in AEVs (143). Interestingly, decreased levels of synaptic proteins synaptopodin and synaptophysin in NEVs were strongly correlated with increased levels of complement proteins (C1q, C3b/iC3b, C5, C5a, fH) in AEVs in MS plasma samples compared to HC (143). Importantly, the increase in complement proteins within EVs was only present in AEVs, while total plasma EVs or neat plasma was not showing differences between MS and HC (143).

### 4.6 Complement-containing EVs in glioblastoma multiforme

Also in the cancer field, circulating EVs may carry potential biomarkers that could be highly valuable to accelerate and further improve the diagnosis and follow-up process for specific malignancies. In this review, we particularly focus on GBM brain tumors, the most prevalent, highly malignant glial tumor in the CNS which is frequently diagnosed only in later disease stages and is associated with a poor prognosis (164). Also in this neurological pathology, the intercommunicative role of EVs has been described in the bidirectional crosstalk between the GBM tumor and its microenvironment (165–167). The involvement of the complement system in GBM pathology is also illustrated by multiple reports and is reviewed by others (124, 168). In general, the complement system seems to play a role in several aspects of GB tumorigenesis, such as the maintenance and migration of glioma stem-like tumor niche cells, GB tumor angiogenesis and immune cell cross talk (124). Other key findings include the presence of complement deposits (C1q, C3, TCC, fB) in tumor tissue (169) and altered complement (C1q, fB) levels in serum (169).

For plasma EVs isolated from GBM patients, three independent proteomic analyses all indicated the enrichment for complement protein C3 compared to HC (148–150). Additionally, also enrichment for C1q, C4b and fH were reported in one of the indicated EV proteome studies (148, 149). Next to complement components, enrichment for other inflammatory and coagulation proteins characterize the overall inflammatory signature of GBM-plasma EVs across studies (148, 149). Strikingly, this inflammatory EV phenotype disappeared after tumor resection, which highlights the potential to use this signature to distinguish GBM tumor bearing patients from HC (149). Findings from GBM mouse model studies further support the growing potential of EV-associated complement components in GBM biomarker research. In a longitudinal study, serum samples for EV analysis were collected to monitor disease progression and therapeutic interventions (154). Here, complement proteins (C1rs, C1ra) were present within the list of deregulated proteins in both GBM 12 days after tumor induction (T1) and GBM 21 days after GL261 implantation (T2) (154). A second longitudinal study with a similar set-up, which analyzed the proteome of EVs from serum collected at baseline, pre-symptomatic (T1) and symptomatic stages (T2), detected upregulated levels of C1qa, C1s1 and C1ra in T1 and T2 stages *versus* baseline, while C4b was downregulated during T1 stage compared to baseline (155).

### 4.7 Complement-containing EVs in ischemic stroke

Besides neurodegenerative and neuroinflammatory diseases, complement proteins are also shown to play a role in the pathogenesis of (neuro)vascular disorders such as atherosclerosis and IS (123, 170). On the one hand, the lectin and alternative complement pathways as well as C3a and C5a binding to their receptors, are implicated in secondary brain tissue injury (123, 170). For example, after a cerebral ischemic event, C3aR and C5aR expression was activated on endothelial cells, glial cells and leukocytes, which may promote inflammatory and/or repair processes at ischemic sites by regulating glial cell activation and chemotaxis (171, 172). Also, the therapeutic potential of modulated complement activation has been illustrated by the fact that modulation or inhibition of complement activation can effectively reduce ischemic brain injury (173, 174). Also, increased levels of C1q and the C1r-C1s-C1inhibitor complex are associated with poor cardiovascular outcomes (175, 176). On the other hand, complement factors including C3a and C5a are also important mediators of neurogenesis and neural plasticity during cerebral ischemia (177, 178). For further extensive reading about the involvement of complement in IS, we refer to reviews written by others (123, 170). In literature, the role of EVs in IS and their potential to transfer information about post-IS processes involving tissue damage and repair have been extensively reviewed as well (179–181). Therefore, linking EVs and complement proteins is an interesting strategy for EV-biomarker identification in IS.

For IS, two proteomic studies on blood-derived EVs were conducted (146, 147). In the first Japanese study with small sample size, four proteins among which C1q subunit B and C1r subunit were significantly enriched in serum EVs from patients who developed symptomatic stroke compared to the HC (146). An important remark here is that the serum samples for EV-proteome analysis obtained in this study were collected during regular health check-ups and not nearby the moment of IS itself, and the diagnosis of IS was based on questionnaires which may imply measurement errors. The second study investigated EV proteome profiles of a larger cohort of IS patients, subdivided in subcortical (SC) and cortical-subcortical (CSC) IS patients, in which serum samples were collected within the first 24h post-IS (147). Here, it was discovered that the C1q A chain protein was specifically present in serum EVs as well as serum-derived NEVs from CSC-IS patients (147). Remarkably, C3 was abundantly present in serum EVs and serum-derived NEVs of CSC- and SC-IS patients as well as HC (147).

## 5 Discussion

Despite the high number of studies investigating complement proteins in the blood and CSF of AD and MS patients, there is currently no clear consensus on their clinical applicability as biomarker. Possible explanations for the high variability across studies include small patient cohorts, different patient inclusion/exclusion criteria or diagnostic parameters and the use of various methods to analyze complement content. Indeed, different complement analysis tools with variable sensitivity and specificity are implemented across biomarker studies to measure complement proteins, including western blot, ELISA, proteomics and multi-array immunoassays. Even when the same detection method for complement components was implemented, variation still arises due to the usage of different complement antibodies or the way samples were prepared for analysis. Moreover, sample collection and analysis should be further standardized for biomarker research and application. Overall, a limited number of studies could show a statistically significant correlation between the level of (a) certain complement protein(s) and disease parameter(s), but the concerning correlations are only described by single studies. In the future, it is required to repeat these studies with bigger patient cohorts to confirm these correlations. However, complement dysregulation is definitely not restricted to one particular CNS disease and almost all complement proteins seem to be affected. Therefore, it is unlikely that free complement components alone will be valuable as biomarkers to distinguish between CNS diseases. Nevertheless, they can still be valuable for supporting the diagnostic process, for example by representing a part of the biomarkers that change within a certain disease instead of considering them as secluded biomarkers for disease diagnosis, progression and response to treatment.

Proteins captured inside or at the surface of EVs as biomarkers have several advantages over free proteins. For example, EVs are stable and protect their cargo from degradation. Interestingly, the cellular source of EVs can be determined by detection of cell-specific markers present on or inside EVs. Finally, EVs are carriers of disease specific signatures because they mimic the status of their cell of origin. This is not only an advantage for diagnostic purposes, but also allows to gain more insights into the underlying disease mechanisms. For example, AD biomarkers are difficult to analyze in peripheral biofluids, while brain-derived EVs can be isolated from peripheral sources by making use of EV enrichment *via* cell type specific markers on EVs (*e.g.*, GLAST, L1CAM). However, before being able to implement EVs as clinical biomarker platform, EV isolation and quality control procedures should be further standardized. Moreover, the knowledge about complement-containing EVs is still limited and until today, no effective correlation studies to link complement-EV levels with disease characteristics have been conducted yet. Notably, as most research on complement EV content is based on proteomic analyses, complement proteins can be detected inside EVs. However, it is possible that the measured complement proteins are only associated with the EVs at their outside instead of being a real part of their cargo. In conclusion, shifting the focus to complement-containing EVs as potential biomarkers for neuroinflammatory and neurodegenerative diseases seems very promising, but further research is still needed to reveal its true value.

## Author contributions

All authors contributed to the article and approved the submitted version.
